# Accelerated CO_2_ transport on surface of AgO nanoparticles in ionic liquid BMIMBF_4_

**DOI:** 10.1038/srep16362

**Published:** 2015-11-09

**Authors:** Dahye Ji, Yong Soo Kang, Sang Wook Kang

**Affiliations:** 1 Department of Chemistry, Sangmyung University, Seoul 110-743, Republic of Korea; 2 Department of Energy Engineering, Hanyang University, Seoul 133-791, Republic of Korea

## Abstract

The AgO nanoparticles were utilized for a CO_2_ separation membrane. The AgO nanoparticles were successfully generated in ionic liquid 1-butyl-3-methyl imidazolium tetrafluoroborate (BMIMBF_4_) by favorable interaction between the surface of particles and the counteranion of BMIMBF_4_. The generated AgO nanoparticles were confirmed by TEM, and the average size was 20 nm. Coordinative interactions of dissociated AgO particles with BMIM^+^BF_4_^−^ were investigated by FT-Raman spectroscopy. When the ionic liquid BMIMBF_4_ containing AgO nanoparticles was utilized as a CO_2_ separation membrane, the separation performance was largely enhanced.

Reducing the emission of carbon dioxide is a recent major concern for global environmental issues, especially climate change. Carbon dioxide results from the continuous use of fossil fuels from coal-fired power plants^1^,^2^,^3^,^4^,^5^. To control the CO_2_ concentration in the atmosphere, conventional methods, such as amine scrubbing, have been proposed for CO_2_ capture in post-combustion^6^,^7^,^8^. However, this conventional method has drawbacks such as high cost and inefficiency because of the degradation of the absorbent at high temperature, as well as toxic and harmful effects^9^,^10^. Thus, imidazolium-based ionic liquids have been suggested for the replacement of volatile organic solvents due to low vapor pressure, thermal stability and chemical stability. Furthermore, imidazolium cations with amine group can also chemically capture CO_2_ and increase the solubility of CO_2_^11^,^12^.

Regarding the ability to affect the solubility of gases in ionic liquids, Noble group reported that permeability is often related to solubility. Thus, ionic liquids should be properly selected for use in gas separation, suggesting trends in the solubility of CO_2_ according to the regular solution theory^12^,^13^,^14^,^15^,^16^,^17^.

Unfortunately, the viscosities of ionic liquids are much greater than those of traditional organic solvents. Furthermore, ionic liquid-based membranes for gas separation have shown a relatively thick selective layer. Because of these characteristics, ionic liquids limit the diffusivity and solubility of gases, resulting in low permeance^18^.

Therefore, to enhance the CO_2_ solubility and gas separation performance in ionic liquids, a carrier that can be reversibly complexed with CO_2_ has been introduced^19^,^20^,^21^. For example, separation performance was reported for 1-butyl-3-methyl imidazolium tetrafluoroborate (BMIM^+^BF_4_^−^)/copper nanoparticles (Cu NPs). Imidazolium cations with an amine group and the surface of Cu NPs partially polarized by BF_4_^−^ anions cause an increase in copper–CO_2_ complexation, enhancing the solubility of CO_2_. This improved separation performance resulted in a CO_2_ permeance of 25 GPU (1 GPU = 1 × 10^−6^ cm^3^ (STP)**/**(cm^2^·s·cm Hg)) and the ideal selectivity of 11 for CO_2_/N_2_^22^.

In the present study, carbon dioxide dissociated in ionic liquids was investigated to increase the solubility. Dissociated silver(II) oxide particles have an strong affinity for CO_2_ because of the oxide layer. As a result, silver(II) oxide as a carrier could be used to selectively remove CO_2_. Imidazolium ions with the amine moiety covalently coupled in BMIM^+^BF_4_^−^ also plays an important role in the increased solubility of carbon dioxide. Thus, when silver(II) oxides are added, ‘free’ ionic liquids are expected to reversibly interact with CO_2_ molecules, increasing the solubility of CO_2_ and resulting in an increase in the separation performance.

## Results and Discussion

**Figure 1:**
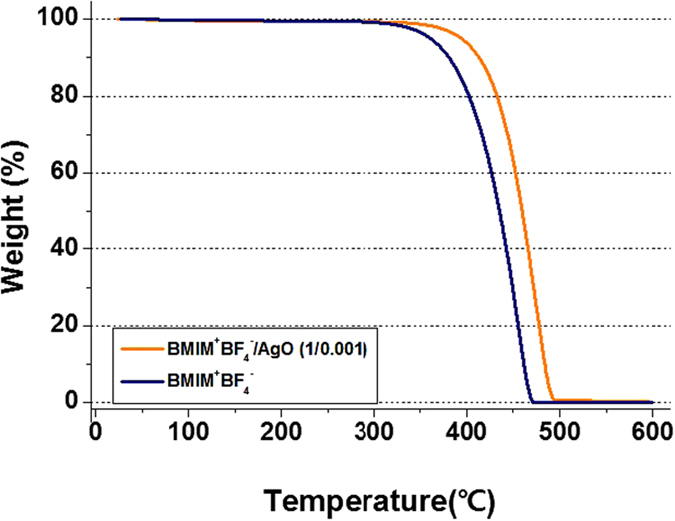
TGA curves of BMIM^+^BF_4_^−^ and BMIM^+^BF_4_^−^/AgO.

The decomposition characteristics of the BMIM^+^BF_4_^−^/AgO composite were assessed by TGA and are shown in Fig. 1. The weight loss of BMIM^+^BF_4_^−^ occurred at 315–470 °C. The prepared BMIM^+^BF_4_^−^/AgO composite was stable up to 345 °C and decomposed at 345–495 °C. The increase of decomposition temperature could be explained by the state of AgO particles in ionic liquid. If the aggregation of AgO particles proceeded in ionic liquid, the decomposition temperature would remain constant. Thus, this phenomenon suggested that the AgO particles were well dispersed in BMIM^+^BF_4_^−^, and the interaction between BMIM^+^BF_4_^−^ and the AgO surface increased the thermal stability of BMIM^+^BF_4_^−^.

**Figure 2:**
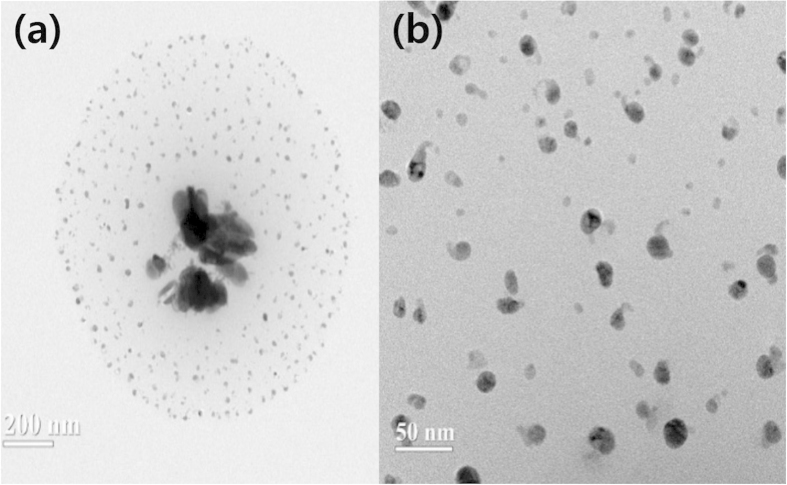
Transmission electron micrographs of (a) the dissociated AgO particles by the ionic liquid and (b) enlarged image. Weight ratio of BMIM^+^BF_4_^−^/AgO = 1/0.001.

TEM images were used to investigate the size and dispersity of the dissociated silver oxide particles, as shown in Fig. 2. Most of the AgO particles were aggregated. However, after sonication, AgO particles were mostly observed as relatively small particles as average 20 nm. It could be expected that these dissociated AgO particles were beneficial for increasing the solubility of CO_2_.

**Figure 3:**
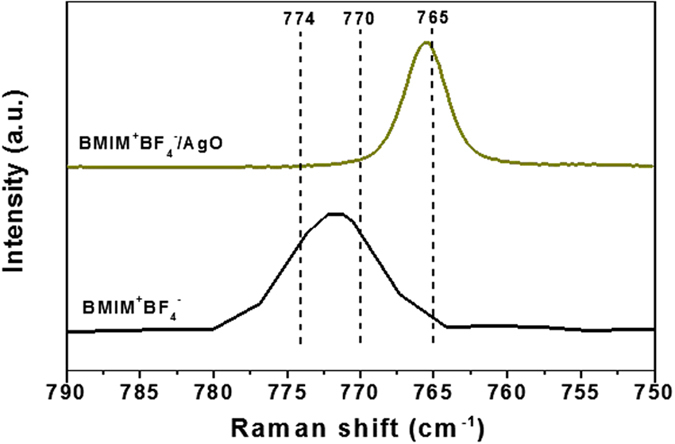
FT-Raman spectra of neat BMIM^+^BF_4_^−^ and BMIM^+^BF_4_^−^/AgO in the BF_4_^−^ stretching region.

Coordinative interactions of dissociated AgO particles with BMIM^+^BF_4_^−^ were investigated by FT-Raman spectroscopy. The Raman spectra in the regions of the BF_4_^−^ stretching bands for the BMIM^+^BF_4_^−^/AgO (1/0.001) are shown in Fig. 3. The BF_4_^−^ bands at 765, 770 and 774 cm^−1^ were assigned to free ions, ion pairs and ion aggregates, respectively. Compared to neat BMIM^+^BF_4_^−^, the symmetric stretch modes at 765 cm^−1^ in free BF_4_^−^ anions increased upon addition of AgO particles. These results could be explained by the weakened interaction between BF_4_^−^ and BMIM^+^. Consequently, the numbers of ion pairs and aggregates decreased. The change in Raman spectra was thought to be due to well-dissociated AgO particles mutually interacting with ionic liquid BMIM^+^BF_4_^−^.

**Figure 4:**
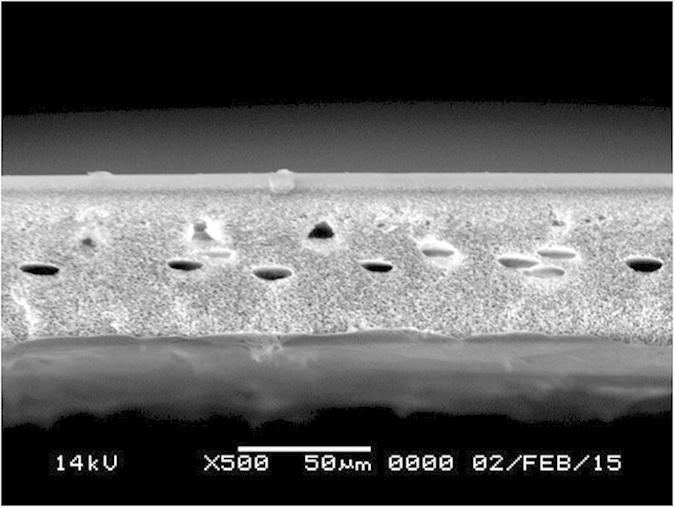
SEM cross-sectional image of the polysulfone support.

The gas permeation properties were different depending on the support. The cross-section structures of the support were investigated by SEM images. A polysulfone support with a structure with a finger-like cross-section was used in our previous reports. However, in the present study, a sponge-like structure was used, as shown in Fig. 4. Compared to the previous finger-like support, the gas permeation performance of the sponge-like structure was relatively low because of conformational differences. The flux of N_2_ was dramatically reduced.

**Figure 5:**
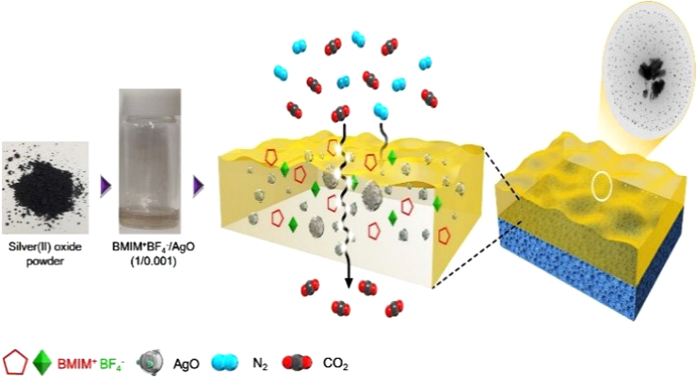
BMIM^+^BF_4_^−^/AgO membrane coated on the polysulfone support (sponge-like structure) for CO_2_ separation.

The dissociated AgO particles in the ionic liquid BMIM^+^BF_4_^−^ were applied to CO_2_ separation. The measurements were done 5 times and the separation performance for CO_2_ permeance and selectivity (CO_2_/N_2_) is shown in Table 1. Neat BMIM^+^BF_4_^−^ and BMIM^+^BF_4_^−^/AgO composite membranes were investigated. Previous study showed that the selectivity of CO_2_/N_2_ and CO_2_ permeance for neat BMIM^+^BF_4_^−^ were 5.0 and 17 GPU^22^. The difference for separation performance was attributable to the different polymer support. Previous study was investigated with polysulforne support to have finger-like structure while this research was done on sponge-like structure. Thus, sine the ionic liquid could be easily penetrated into polymer support, the possibility of defect between support and liquid was high. Therefore, the relatively selectivity was lower and peremance was higher for than finger-like than sponge-like structure. When AgO was dissociated into the ionic liquid BMIM^+^BF_4_^−^, the separation performance was significantly enhanced. These enhanced values could be compared with previous membranes consisting of BMIM^+^BF_4_^−^/Cu nanoparticles^22^. BMIM^+^BF_4_^−^/Cu nanoparticles composite membranes showed the selectivity of 11 and 25 GPU for CO_2_^22^. The enhanced selectivity of BMIM^+^BF_4_^−^/AgO than BMIM^+^BF_4_^−^/Cu nanoparticles composite membranes was attributable to the interaction between the oxide layer of the dissociated AgO particles and CO_2_. As another example, poly(ethy1ene oxide) (PEO) containing a polar ether group was reported to increase the solubility of CO_2_ due to an affinity for CO_2_^23^,^24^,^25^,^26^,^27^. Thus, CO_2_ is better able to penetrate through PEO than is N_2_. Therefore, the oxide layer of the dissociated AgO affects the increase in CO_2_ solubility. Furthermore, free imidazolium cations in ionic liquids interact with AgO to play a role as CO_2_ carriers for facilitated transport, resulting in enhanced solubility and diffusivity of CO_2_. As a result, BMIM^+^BF_4_^−^/AgO composite membranes were significantly improved compared with neat BMIM^+^BF_4_^−^, as shown in Fig. 5. The ideal selectivity for CO_2_/N_2_ was 28.2 with a CO_2_ permeance of 14.1, while the neat BMIMBF_4_ membrane showed a selectivity of 8.8 and a CO_2_ permeance of 5.3.

In conclusion, the ionic liquid BMIMBF_4_/AgO composite membrane was successfully prepared for CO_2_ separation. When the AgO nanoparticles were generated in ionic liquid BMIMBF_4_ by strong interaction between the surface of the particles and counteranion of the ionic liquid, the selectivity of CO_2_/N_2_ and CO_2_ permeance were largely enhanced to 28.2 and 14.1, respectively, while the neat BMIMBF_4_ membrane showed a selectivity of 8.8 and CO_2_ permeance of 5.3. These enhancements in both selectivity and permeance were attributed to a synergy effect: (1) the oxide layer of the dissociated AgO affects the increase in CO_2_ solubility, and (2) free imidazolium cations in ionic liquids interact with AgO to play a role as CO_2_ carriers for facilitated transport.

## Methods

### Materials

1-Butyl-3-methyl imidazolium tetrafluoroborate (BMIM^+^BF_4_^−^) was purchased from Merck KGaA (Darmstadt, Germany). AgO was purchased from Sigma-Aldrich Chemical Co. Ethyl alcohol (greater than 94.0%) was purchased from Daejung Chemicals & Metals. All initial solvents and materials were used as received.

### Fabrication process

The membranes were prepared using BMIM^+^BF_4_^−^, silver(II) oxide and ethanol. First, silver(II) oxide was sonicated to disperse in ethanol for 5 min. Then, BMIM^+^BF_4_^−^ was added to the mixtures of silver(II) oxide and ethanol. The solution was heated at 70 °C under constant stirring to evaporate the ethanol. Since the BMIM^+^BF_4_^−^ was liquid state, the porous polymer support was utilized. Thus, the final solution was coated onto a macroporous polysulfone support to have average 0.1 *μ*m pore (Toray Chemical Korea Inc.) and then cast using a RK control coater (Model 101, Control Coater RK Print-Coat Instruments Ltd., UK). The best performance of BMIM^+^BF_4_^−^/silver(II) oxide was observed at 1/0.001 (weight ratio). Gas permeance values were measured with a bubble flow meter at upstream 2 kgf·cm^−2^ and atmospheric downstream pressure. Gas permeance is expressed as units of GPU (1GPU = 1 × 10^–6^ cm^3^ (STP)/(cm^2^ s cmHg)).

### Characterization

A sonifier (Branson 450, Branson Ultrasonics Corporation, Danbury CT, USA) with a standard tip was used. Thermogravimetric analysis (TGA) was obtained using a Universal V4.5A TA. The TEM images were obtained using a JEOL JEM-3000 operating at 300 kV. Raman spectra were obtained using a Horiba Jobin–Yvon/LabRAM ARAMIS instrument at 785nm (diode laser). The structural morphology was observed by scanning electron microscopy (SEM, JEOL JSM-5600LV, Japan).

## Additional Information

**How to cite this article**: Ji, D. *et al.* Accelerated CO_2_ transport on surface of AgO nanoparticles in ionic liquid BMIMBF_4_. *Sci. Rep.* **5**, 16362; doi: 10.1038/srep16362 (2015).

## Figures and Tables

**Table 1:** Permeance and selectivity of neat BMIM^+^BF_4_^−^ and BMIM^+^BF_4_^−^/AgO (1/0.001, weight ratio).

	Permeance (GPU)		Selectivity
	N_2_	CO_2_	CO_2_/N_2_
Neat BMIM^+^BF_4_^−^ 22	3.4	17	5.0
BMIM^+^BF_4_^−^/Cu^22^	2.3	25	11
Neat BMIM^+^BF_4_^−^	0.6 ± 0.02	5.3 ± 0.2	8.8
BMIM^+^BF_4_^−^/AgO	0.5 ± 0.02	14.1 ± 0.2	28.2
